# Verification Index for the Repositioning of a Single Implant Analog in the Correct Place: A Clinical Technical Case Report

**DOI:** 10.3390/reports9030288

**Published:** 2026-08-27

**Authors:** Socratis Thomaidis

**Affiliations:** Department of Operative Dentistry, National and Kapodistrian University of Athens, 11527 Athens, Greece; sthom@dent.uoa.gr

**Keywords:** dental implants, impression accuracy, misfit, transfer jig, repositioning jig, transfer index, repositioning index, clinical case report

## Abstract

**Background and Clinical Significance**: Implant impressions can present inaccuracies, affected by many factors, such as impression technique, impression material, and parallelism or lack thereof among the implants. The use of a verification index can assess the accuracy of the mastercast and can be used in order to adjust the inaccuracy of the mastercast. This article presents a technical variation in established verification-index procedures, followed by implant analog repositioning, which can be used in case of an impression inaccuracy; **Case Presentation**: A patient with a moderate gag reflex received an implant. The impression of an implant and a prepared tooth was made. At the metal try-in, an inaccuracy of the mastercast was found, attributed to the final impression. A technique was illustrated, describing a polymethyl methacrylate (PMMA) verification index fabrication, followed by implant analog repositioning in the removable die of the master cast with the use of acrylic resin. This method may be used as an alternative to repeating the impression in selected clinical situations, but it is time-consuming, technique-sensitive, and needs meticulous handling; **Conclusions**: This is a viable technique and may represent an alternative to repeating the impression in selected clinical situations. Therefore, the clinical workflow can be reduced by one appointment. It can be helpful for patients with a moderate to severe gag reflex, since it can minimize the discomfort and stress for the patient and the dentist.

## 1. Introduction and Clinical Significance

Implant-supported crowns and bridges can predictably restore partially or fully edentulous ridges [1,2]. The fit of implant screw-retained partial dentures is of paramount significance for their survival, and a misfit range of up to 150 μm is considered acceptable [3,4,5,6,7,8,9]. Misfit at the implant–prosthesis interface arises from small inaccuracies occurring during impression-making [10], casting [11] and milling [12]. When an inaccuracy is identified in an implant-supported prosthesis, the use of a verification index can be made in order to assess and record it. Subsequently, the verification index can be used to adjust the inaccuracy of the mastercast.

Passive fit of the implant restoration is very important for its survival. Failure to achieve passive fit has been associated with the development of peri-implantitis and loss of peri-implant tissue at the apex of the apical ridge. It can also be associated with prosthetic complications such as loosening or fracture of the prosthetic screw, fracture of the transmucosal abutment, chipping of the veneering ceramic material, or fracture of the restoration [13,14,15,16,17,18].

Screw-retained implant-supported prostheses have the advantage of retrievability, while cement-retained multi-unit restorations can present a passive fit [19]. When the implant position and/or angulation is not favorable for screw-retained restorations, an angulated or cast abutment can be used, with a cemented restoration [20,21]. Angled screw access abutments can also be used [22]. The open tray impression technique is considered more accurate than the closed tray technique [18,23,24,25,26]. It has also been reported that a closed tray implant impression can be as accurate, or even more accurate, than an open tray impression [27,28]. The accuracy of implant impressions can be affected by the parallelism of implant placement [25,29,30,31], the depth of the implant position [32], the type of impression material used [26,33,34], the dimensional stability of the stone used to fabricate the cast [35], the die system used [36], and the length of the impression copings [37]. Some investigators reported that the single-stage double-mix technique leads to more accurate impressions compared to the two-stage technique [38,39], while others reported that the two-stage technique was more accurate [40] or that no statistically significant differences were detected among these techniques [41,42]. However, sometimes inaccuracies occur in implant impression making. Then a new impression is needed. Instead, a verification index and die repositioning techniques in dental mastercasts have been reported for fixed partial dentures, supported by teeth and/or implants [43]. The optimum verification index should be rigid, dimensionally stable, easy to construct, and not contact the soft tissues [43]. Numerous materials have been used for the fabrication of a verification index, such as poly-methyl methacrylate (PMMA) [44,45,46,47,48], PMMA sectioned and reconnected [49], PMMA in connection with metal [50], molding tray material [51], light polymerizing tray material [52], modeling compound [53], and stone grade IV [54]. A technique using multiple interlocking puzzle pieces and engaging interim abutments has been reported in order to verify the 3-dimensional spatial orientation of implant analogs on definitive casts [55]. The use of multi-unit abutment handles in conjunction with 3D-printed material has also been proposed [56]. Verification indexes are also advocated in the digital workflow of implant-supported restorations, with the use of PMMA and a 3D-printed surgical guide [57]. Verification indexes, made out of PMMA, are more practical and easier to fabricate than the ones made out of dental stone. Photopolymerizing composite resins used for verification index fabrication tend to distort [43]. Die repositioning in matercasts with removable dies, intended for fixed prosthodontics, has been performed with the use of dental stone [45], acrylic resin [44], or cyanoacrylate cement [52]. Implant analog repositioning, in solid mastercasts, has been performed with the use of dental stone [48,50].

The gag reflex is a normal defense mechanism that prevents foreign bodies from entering the trachea, pharynx, or larynx. The gag reflex is a prevalent clinical challenge in dental practice that complicates making dental impressions and may potentially lead to treatment avoidance [58,59,60].

The hard stone presectioned mastercast can present a dimensional change of 0.11% due to the setting expansion of hard dental stone [61]. When a sectioned die system mastercast is fabricated, an accuracy of 0.124% is expected, with the Pindex system presenting the highest accuracy when compared to the Belle de St. Claire system and conventional brass dowel pin system, with a dimensional change of 0.056% [61]. The Zeiser system, used for mastercast fabrication, is considered more accurate than a solid cast [62].

Metal-ceramic restorations are considered to present higher values of mechanical properties than translucent monolithic zirconia and glass ceramics [63,64,65,66,67,68,69] but demonstrate inferior translucency and esthetics [68,69].

The aim of this clinical technical case report was to illustrate clinical and laboratory procedures used in order to verify the position of an implant and reposition an inaccurate implant analog in a master cast with removable dies for a patient with a moderate gag reflex. The analog repositioning, within the removable stone die, is performed with the use of acrylic resin. This technique may represent an alternative to repeating the impression in selected clinical situations.

## 2. Case Presentation

A 70-year-old patient, with a free medical history and moderate gag reflex, presented at the office, complaining about mobility of the maxillary left lateral incisor (#22) as well as poor esthetics of the maxillary left canine tooth (#23). The patient suffered from generalized stage II grade I periodontal disease and was under a 6-month recall by a periodontist.

**Figure 1:**
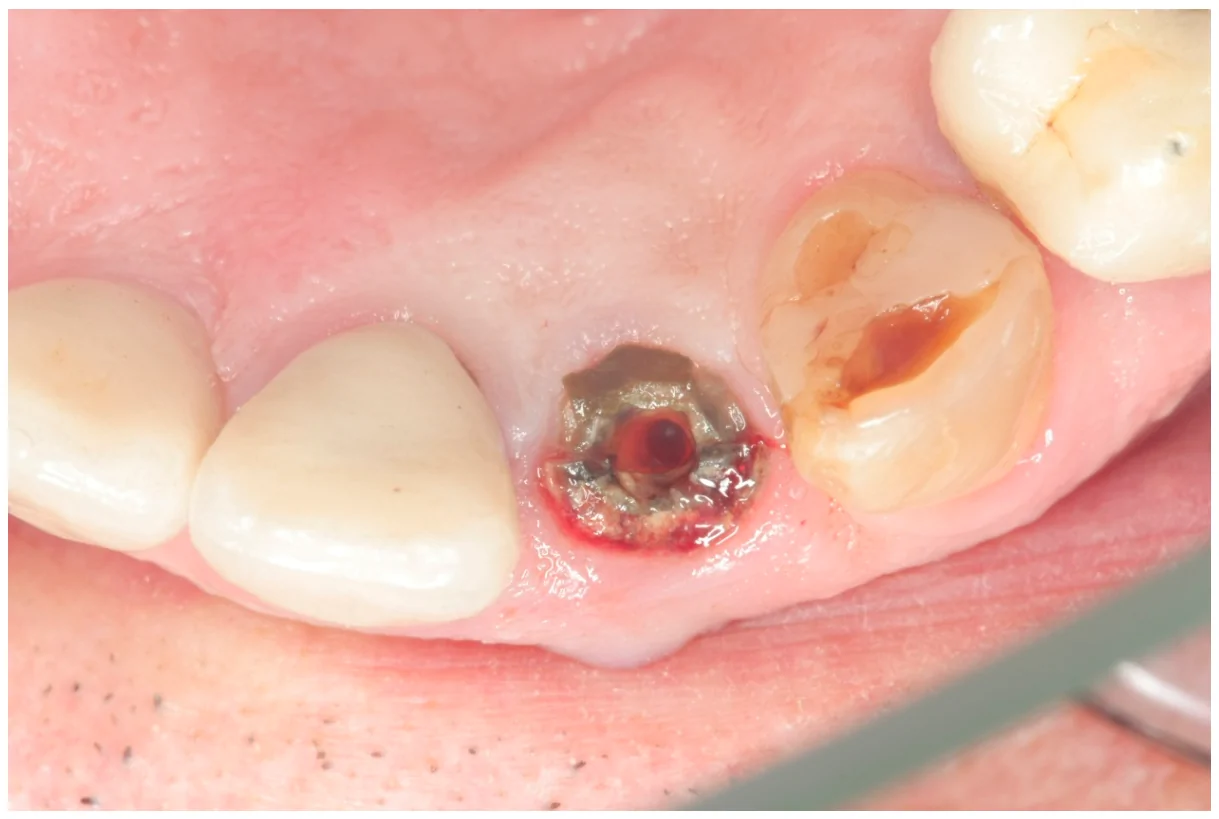
The vertically cracked tooth #22. Initial situation.

**Figure 2:**
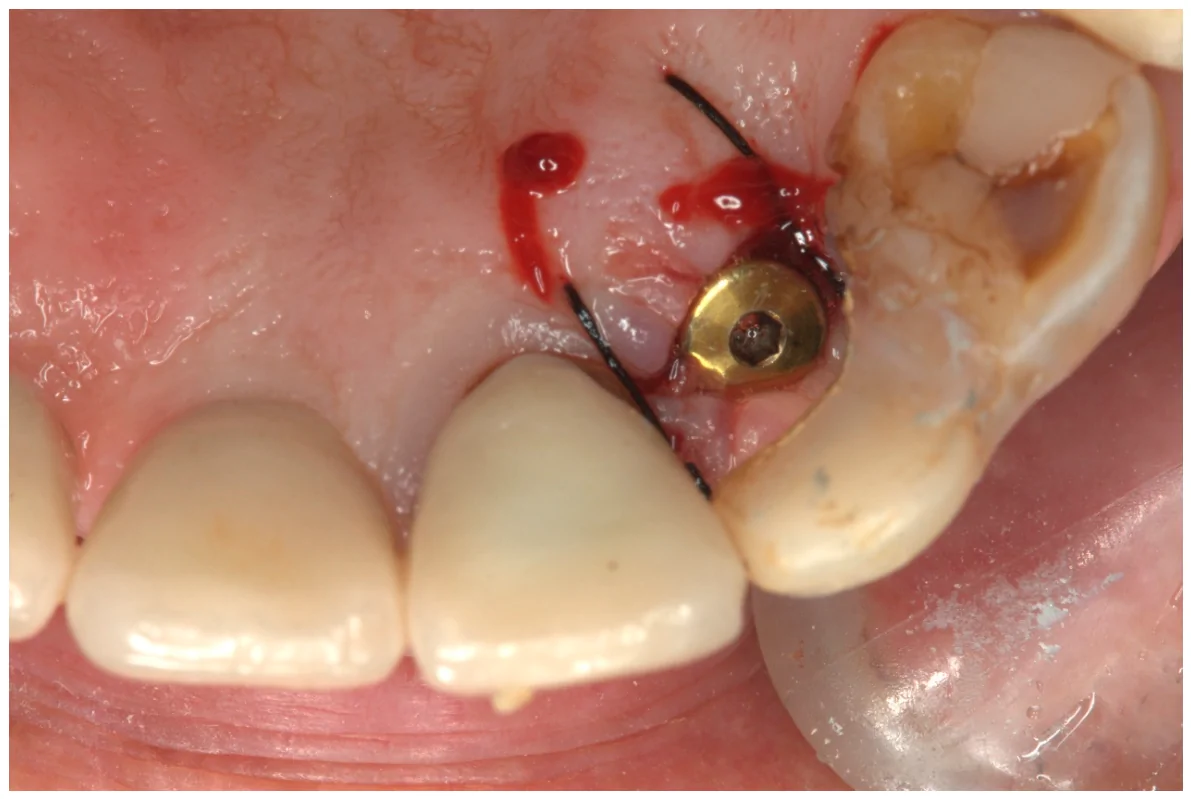
The implant with a healing abutment and a provisional restoration bonded to tooth # 23.

The maxillary left lateral incisor (#22) was restored with a porcelain fused to metal (PFM) crown and a post and core. Upon examination, the PFM crown on the maxillary left lateral incisor (#22) came loose with a cast post and core attached to it, and a vertical fracture was evident in the supporting root (Figure 1). The patient was informed that the tooth had to be extracted. Maxillary right central and lateral incisors (#11 and 12) were previously restored with implant-supported cemented crowns. The maxillary left central incisor (#21) was restored with a PFM crown, presenting overeruption, but the patient refused to have it replaced with a 3-unit bridge. A delayed implant insertion protocol was selected, and the extraction was performed. A composite resin build-up (Clearfil Majesty ES-2 Universal, Kuraray, Okayama, Japan) bonded (Clearfil SE Bond, Kuraray, Japan) to the canine served as an interim restoration. A CBCT (cone beam computed tomography) was made, and a narrow platform internal connection implant (3.3 mm × 10mm MIS seven) was placed (Figure 2). A preliminary alginate impression was made, which was scanned by the dental technician with a laboratory scanner (Aadva Lab Scan, GC Tech, Breckerfeld, Germany) with nominal accuracy < 10 μm. Consequently, a digital model (standard triangle language or standard tessellation language file, STL file) was created. The file from the CBCT (Dicom information) was then aligned to the digital model (STL file) with the use of BlueSky Bio Plan v5.0 software (BlueSky Bio digital) in order to design and print a surgical guide for implant placement. During the design of the implant placement position, with the use of BlueSkyBio software, it was found that the residual bone present was not enough for the placement of the implant in a prosthetically driven position. Moreover, the patient was not willing to wait for bone augmentation procedures prior to implant placement, and thus an implant was planned and positioned in a position capable of receiving a cemented restoration.

An alloplastic bone graft (EthOss, Ethoss Regeneration Ltd., Silsden, UK), comprising 65% β-TCP and 35% calcium sulfate binder, was used buccally, after implant placement, covered with the patient’s periosteum, and sutured. Therefore, no barrier membrane was used. Mucogingival surgery was not performed because the patient refused to accept this treatment plan. Moreover, the patient presented a low smile line. Therefore, the gingival recession was not apparent during smiling. The same interim restoration was used during the osseointegration period as well.

**Figure 3:**
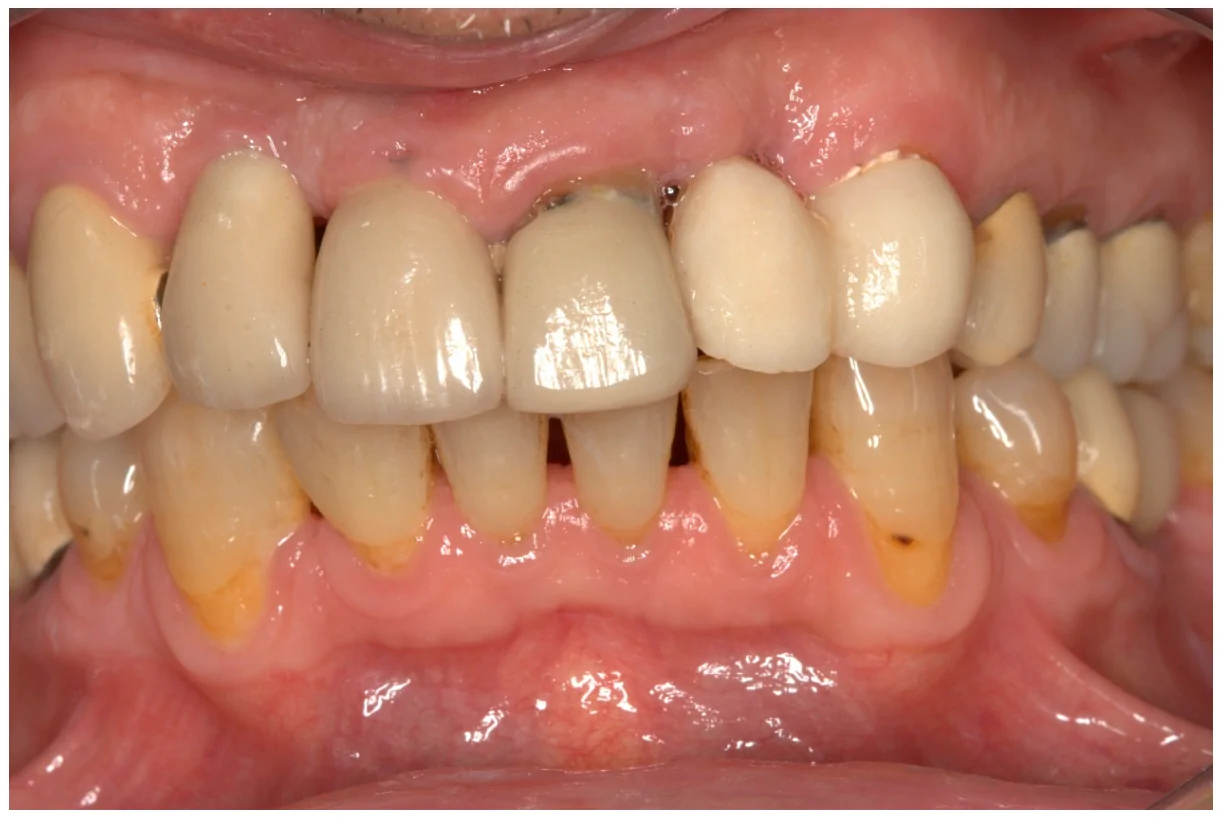
The provisional was placed on tooth #23, with a cantilever for #22, following the osseointegration period.

**Figure 4:**
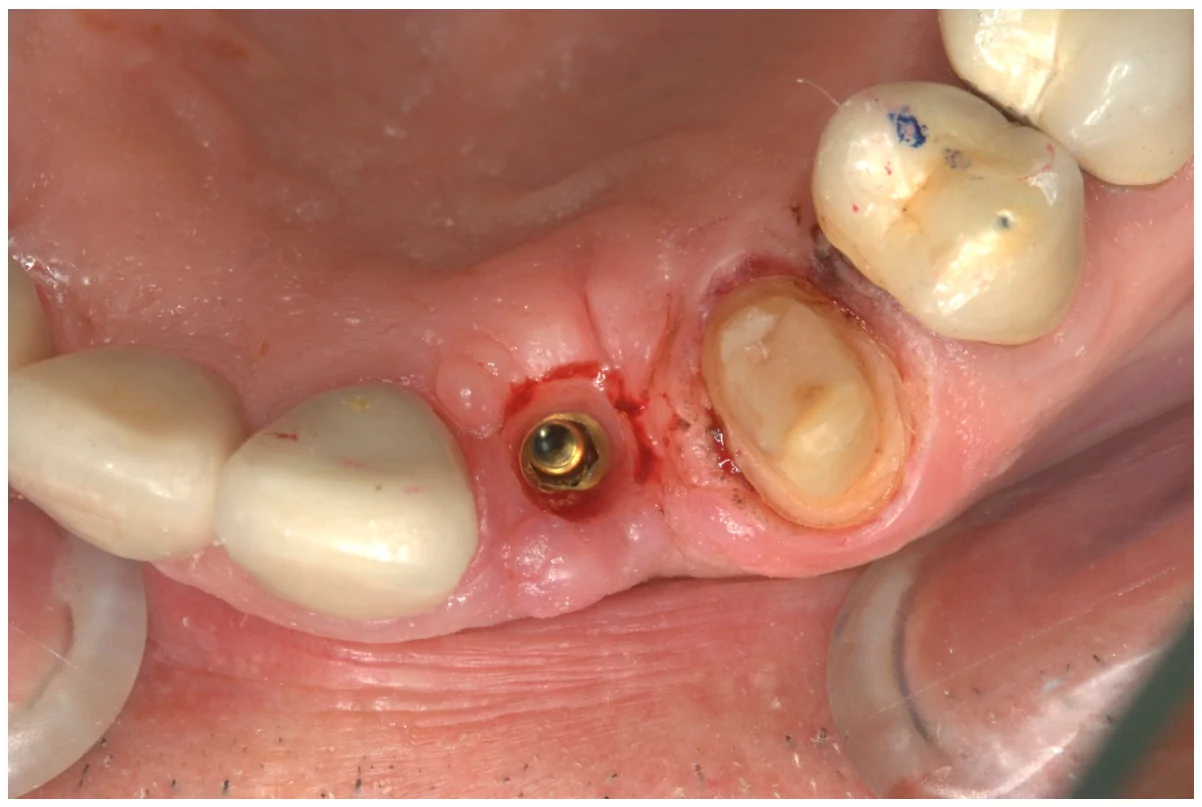
The implant and prepared tooth after impression making.

**Figure 5:**
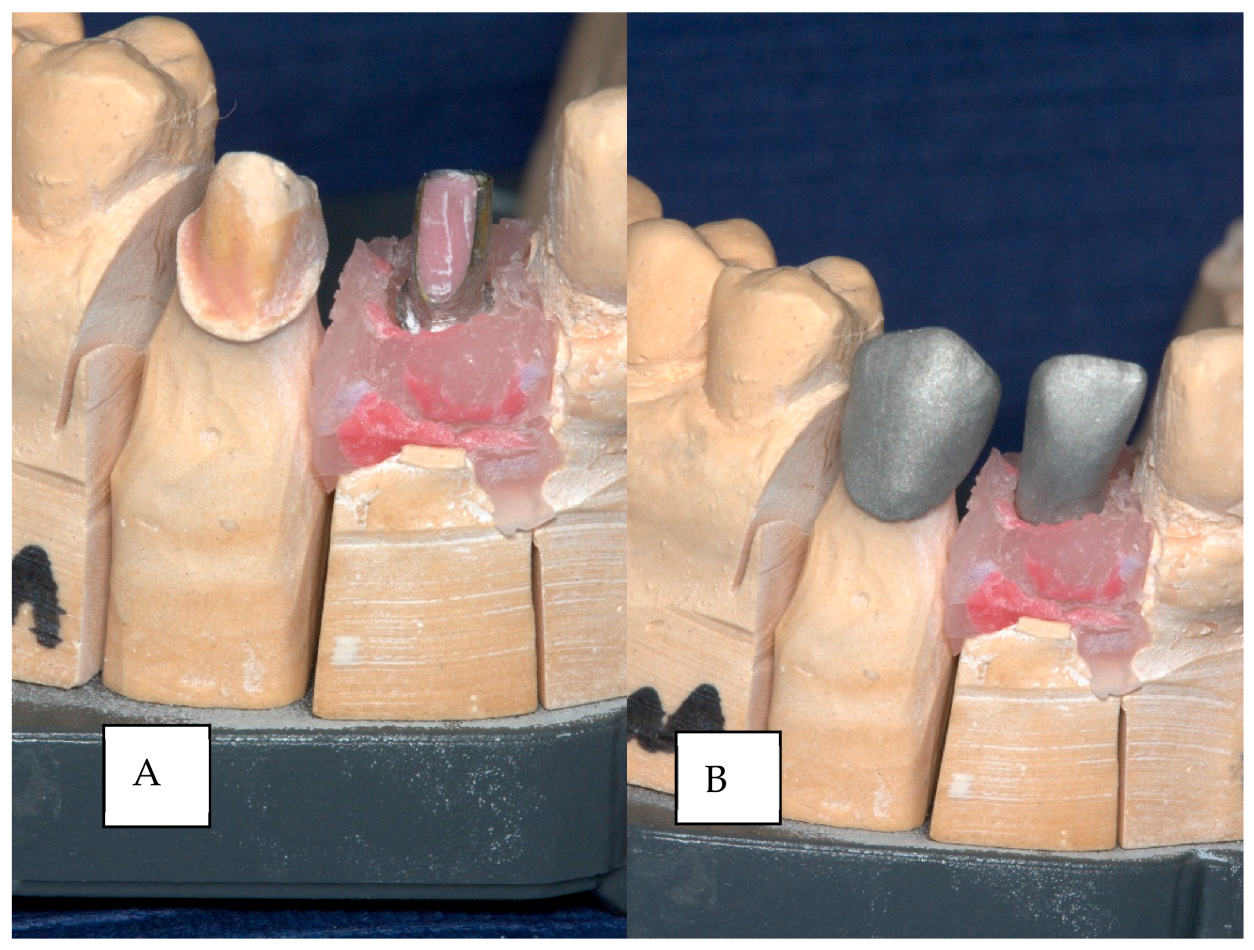
(**A**) The mastercast, with the abutment in place. (**B**) The mastercast with the metal frameworks, before metal try-in.

**Figure 6:**
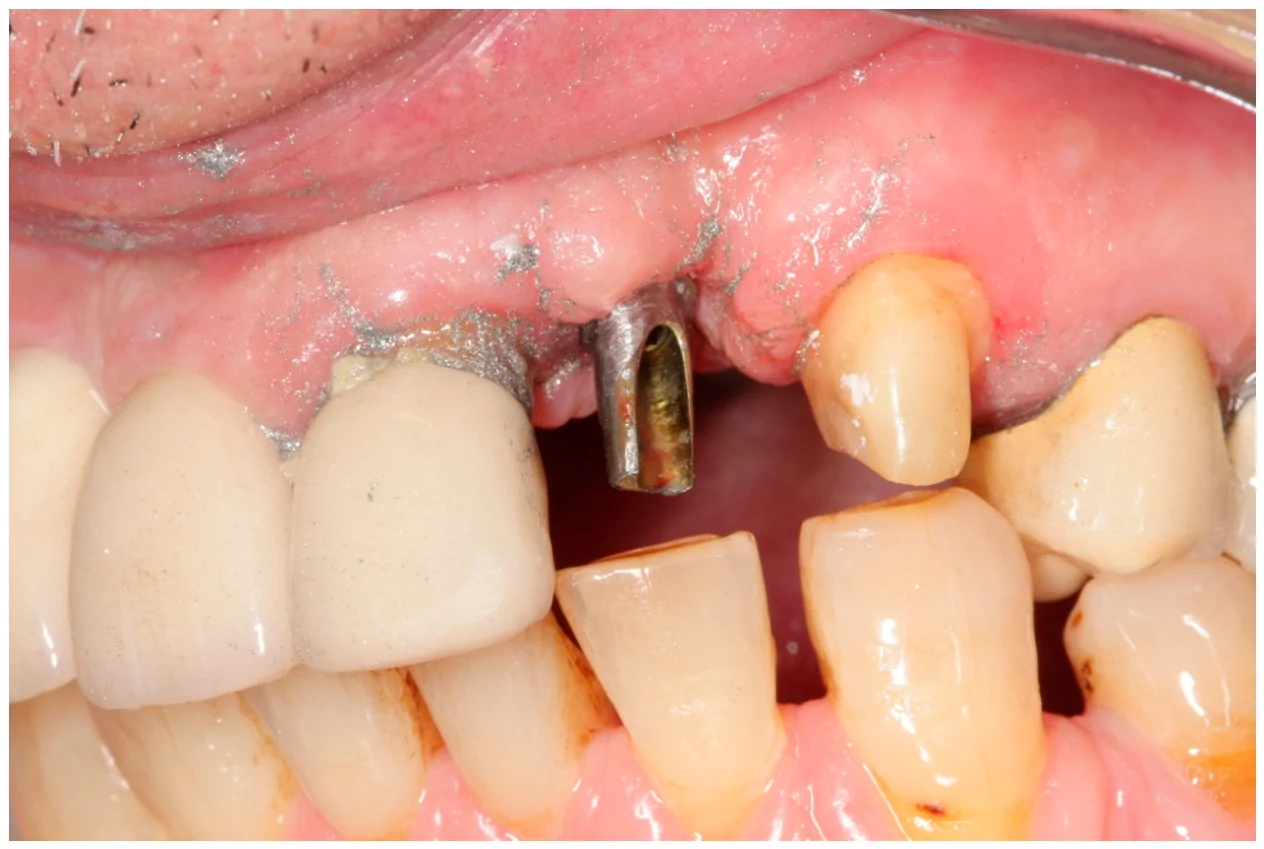
The machined abutment screwed onto the implant.

**Figure 7:**
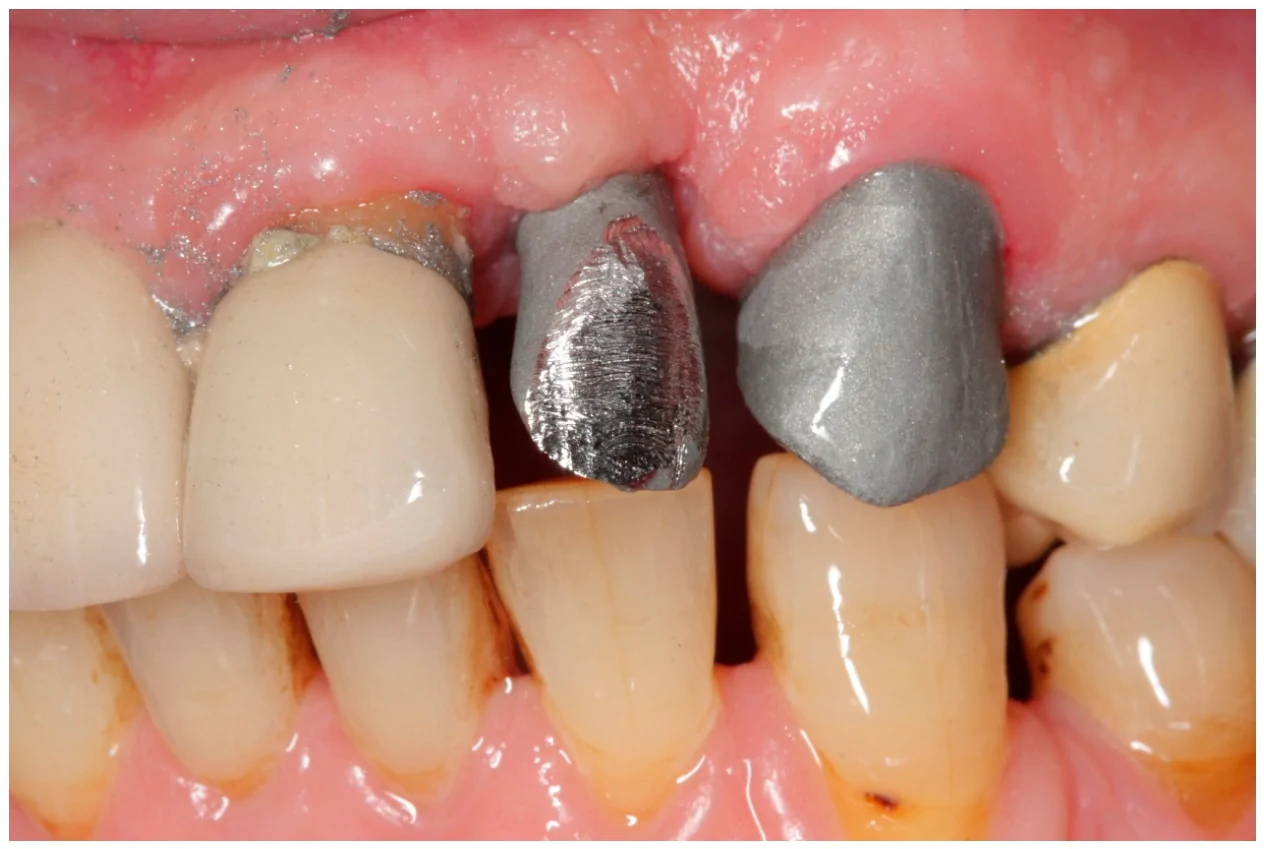
Metal framework try-in after adjustment.

The maxillary left canine (tooth #23) was prepared for a PFM crown, and a chemically cured methyl-methacrylate (Jet tooth shade A3 powder and liquid, Lang, Wheeling, IL, USA) provisional restoration, with a cantilever for tooth # 22, was made and temporarily cemented (Figure 3 and Figure 4). Upon second-stage surgery, a healing abutment was placed, and 15 days later an open tray impression was made for the implant as well as for the prepared canine. A double retraction cord was placed around tooth #23. A perforated stock tray was used, and a double-mix heavy-body/light-body polyvinyl siloxane (Affinis Precious Light Body/Affinis Heavy Body, Coltene, Altstatten, Switzerland) impression was made and sent to the dental lab. The light-body polyvinyl siloxane was used only around the prepared tooth, while the heavy-body was injected around the impression coping. A master cast with removable dies (Zeiser system) was made by the dental technician. A 10^o^ machined abutment for cemented restorations (MIS) was selected and prepared by the dental technician accordingly in order to present a proper path of insertion and enough space for the metal–ceramic restoration. Metal frameworks for teeth #22 and 23 were then fabricated (cast) (Figure 5). Upon the metal try-in appointment, the abutment was screwed onto the implant (Figure 6), and the metal framework did not present the same way as it did on the master cast due to impression inaccuracy (Figure 7).

**Figure 8:**
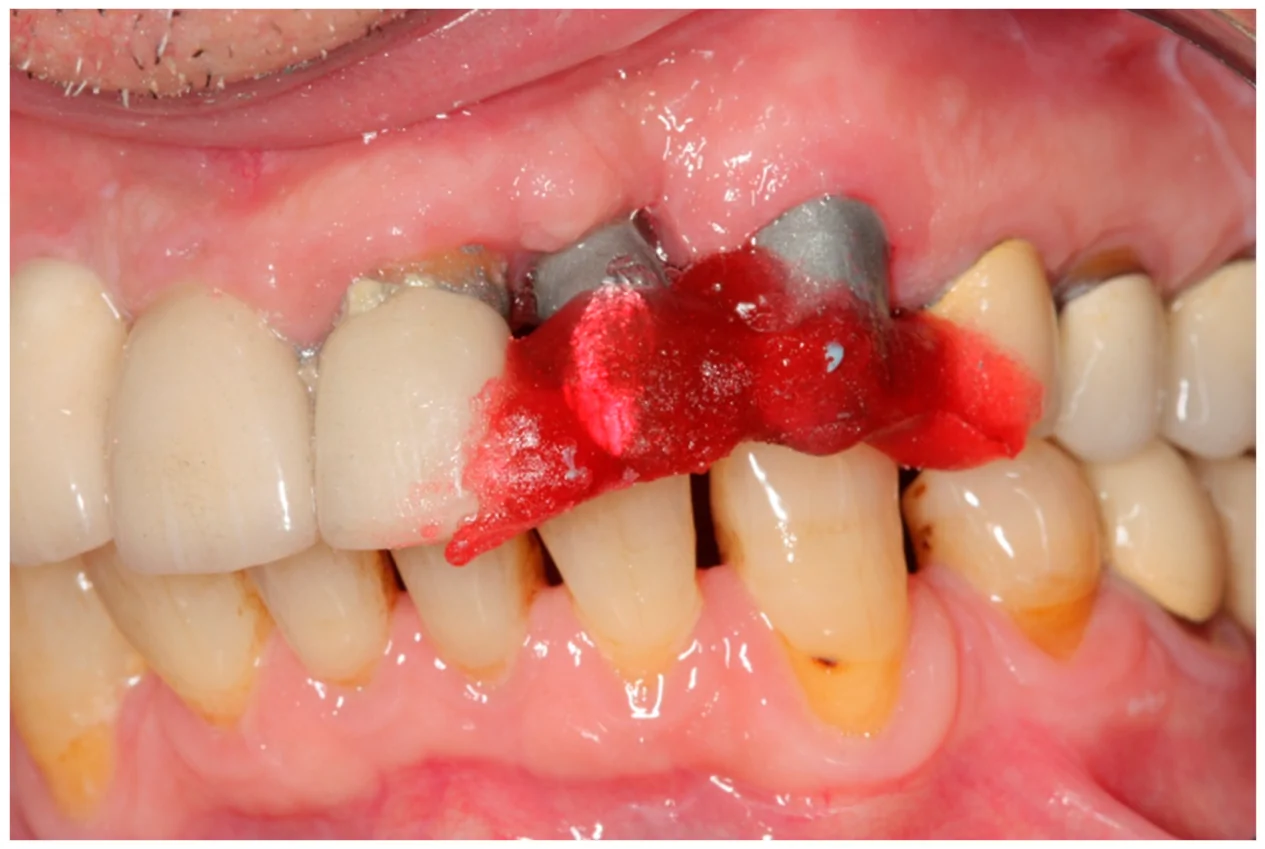
The acrylic verification index with the crowns seated upon the implant abutment and prepared tooth.

**Figure 9:**
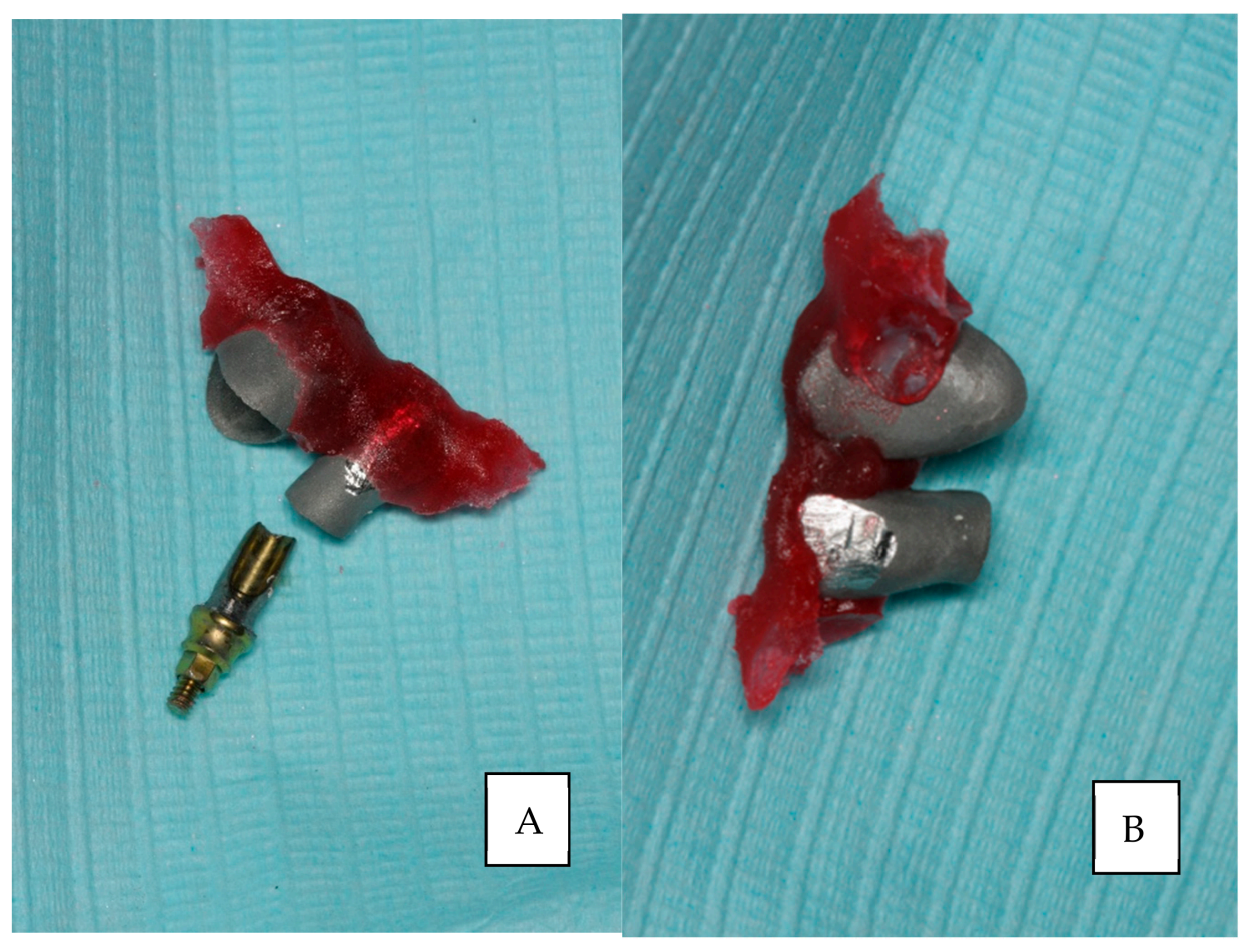
Buccal (**A**) and lingual (**B**) views of the acrylic verification index with the metal frameworks of crowns #22 and #23.

**Figure 10:**
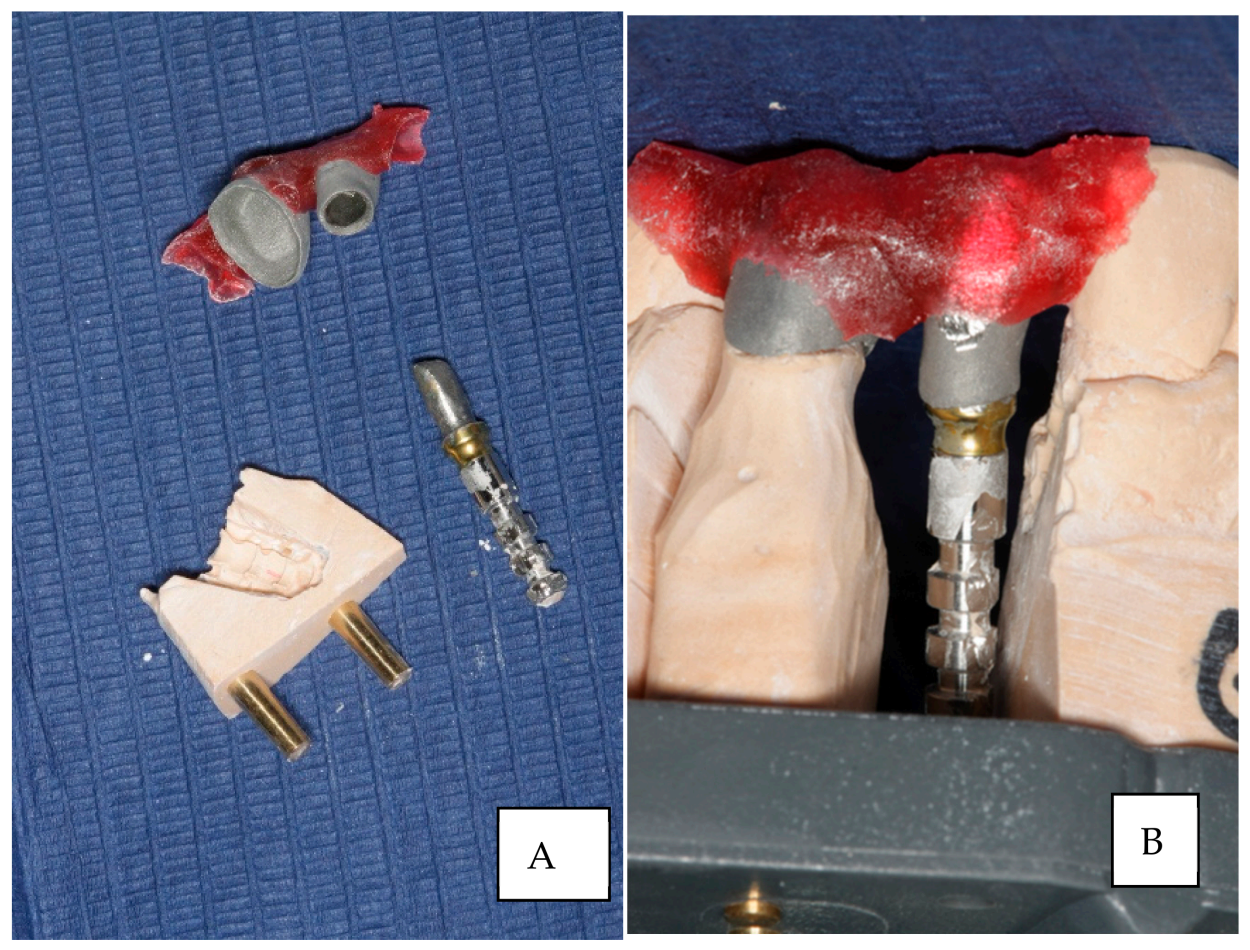
(**A**) The die after removing the implant analog, verification index, and implant analog; (**B**) the implant analog, verification index, and implant analog repositioned upon the mastercast, without the removable die.

**Figure 11:**
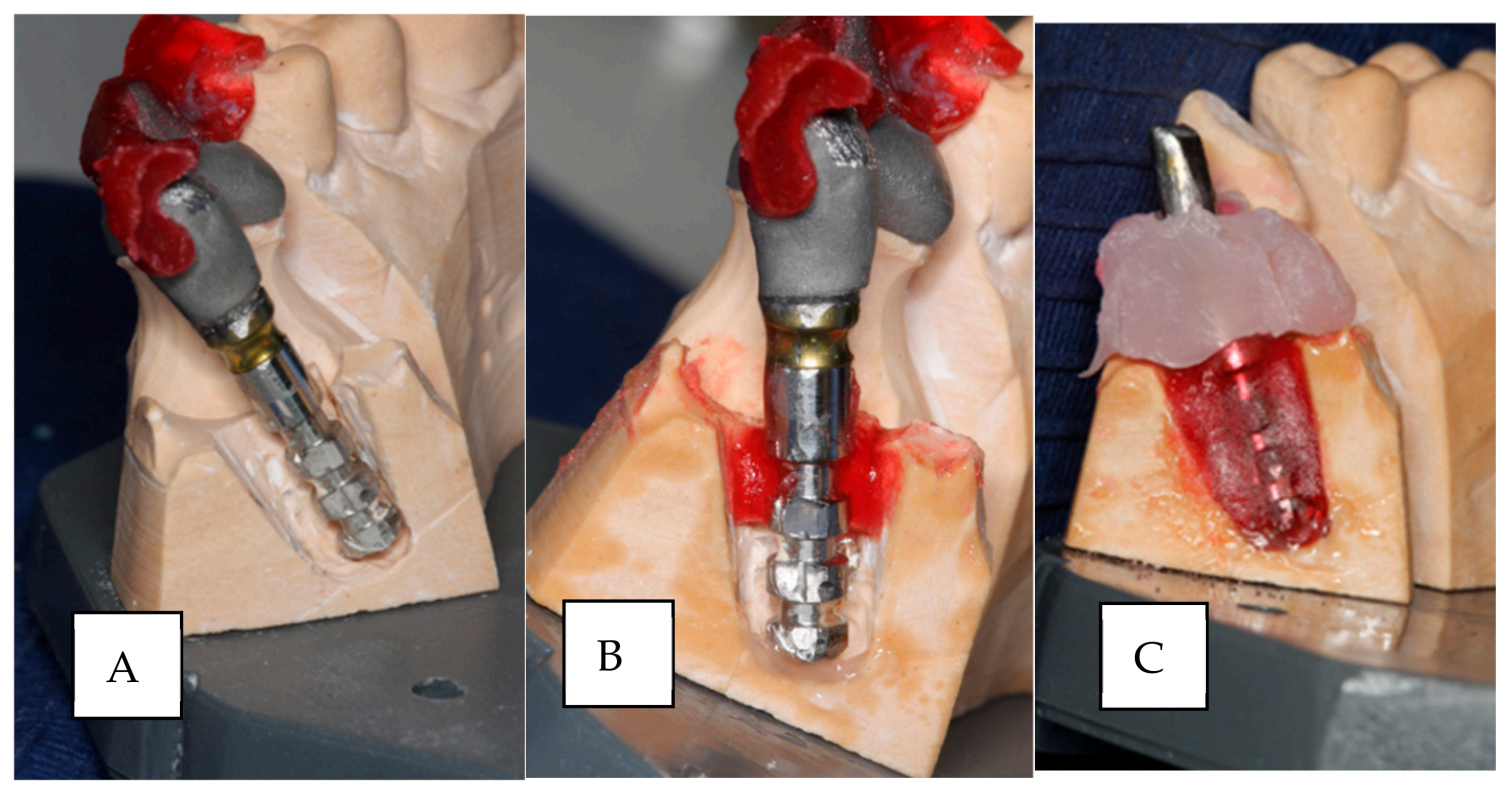
(**A**) Connecting the verification index with the crowns repositioned over the removable die placed upon the mastercast; (**B**,**C**) the connection of the implant analog to the removable die with acrylic resin.

The abutment position was evaluated and was considered acceptable, offering adequate space for the crown fabrication. The space offered by the metal framework was considered enough for a metal–ceramic restoration after some minor adjustment. The extent of the inaccuracy enabled the grinding of the metal framework for the crown on #22 in order to create enough space for properly supporting the veneering porcelain (Figure 7). A depth caliper gauge was used to ensure that the metal framework thickness was not less than 0.3 mm. After verifying that the two castings were properly seated on the two abutments, a verification index was made out of a self-curing methyl methacrylate-based acrylic resin (GC Pattern Resin), which was placed around the metal frameworks and on the adjacent teeth. The castings and adjacent teeth were lubricated with a separating medium (COE Sep tinfoil substitute, GC, Alsip, IL, USA). The index was made surrounding the metal frameworks of the crowns and covering the adjacent teeth above their maximum circumference in order not to lock around them. When the acrylic index was set, it was sectioned between the castings on 22 and 23, then the two parts were connected with new acrylic and left to set for 5 min. Afterward, the fit was evaluated with the use of 3.5X magnification dental loupes (Figure 8). Upon acrylic setting, the index was removed, relieved on the interior surface of the indentations, and relined in order to minimize contraction of the acrylic (Figure 9). Provided that the fit of the acrylic index is not good enough, the procedure should be repeated. The removable die, containing the implant analog, was ground with the use of a small bur in the airotor handpiece in order to remove the implant analog (Figure 10A). Adequate space between the new position of the implant analog and the adjacent removable dies (Figure 10B), as well as with the ground die, was provided (Figure 11A). The abutment was screwed onto the implant analog. A separating solution was applied to the adjacent areas of the mastercast, as well as on the removable die around its pin, and the removable die was placed on the mastercast. Subsequently, the index was attached to the castings of #22 and 23 with sticky wax. Afterward, the machined abutment, with the attached analog, was secured on the casting of #22 with sticky wax. The whole assembly was then meticulously placed on the master cast and was seated on the die of tooth #23. At this point, it should be verified that enough space is created in the removable die in order not to contact the implant analog (Figure 11A). The space between the prepared stone die and implant analog was secured with a self-curing methyl methacrylate-based acrylic resin (Pattern Resin LS, GC, Alsip, IL, USA) and left to set (Figure 11B). Subsequently, the removable die was removed from the mastercast and filled with self-curing methyl methacrylate-based acrylic resin (Pattern Resin LS, GC, USA) (Figure 11C).

**Figure 12:**
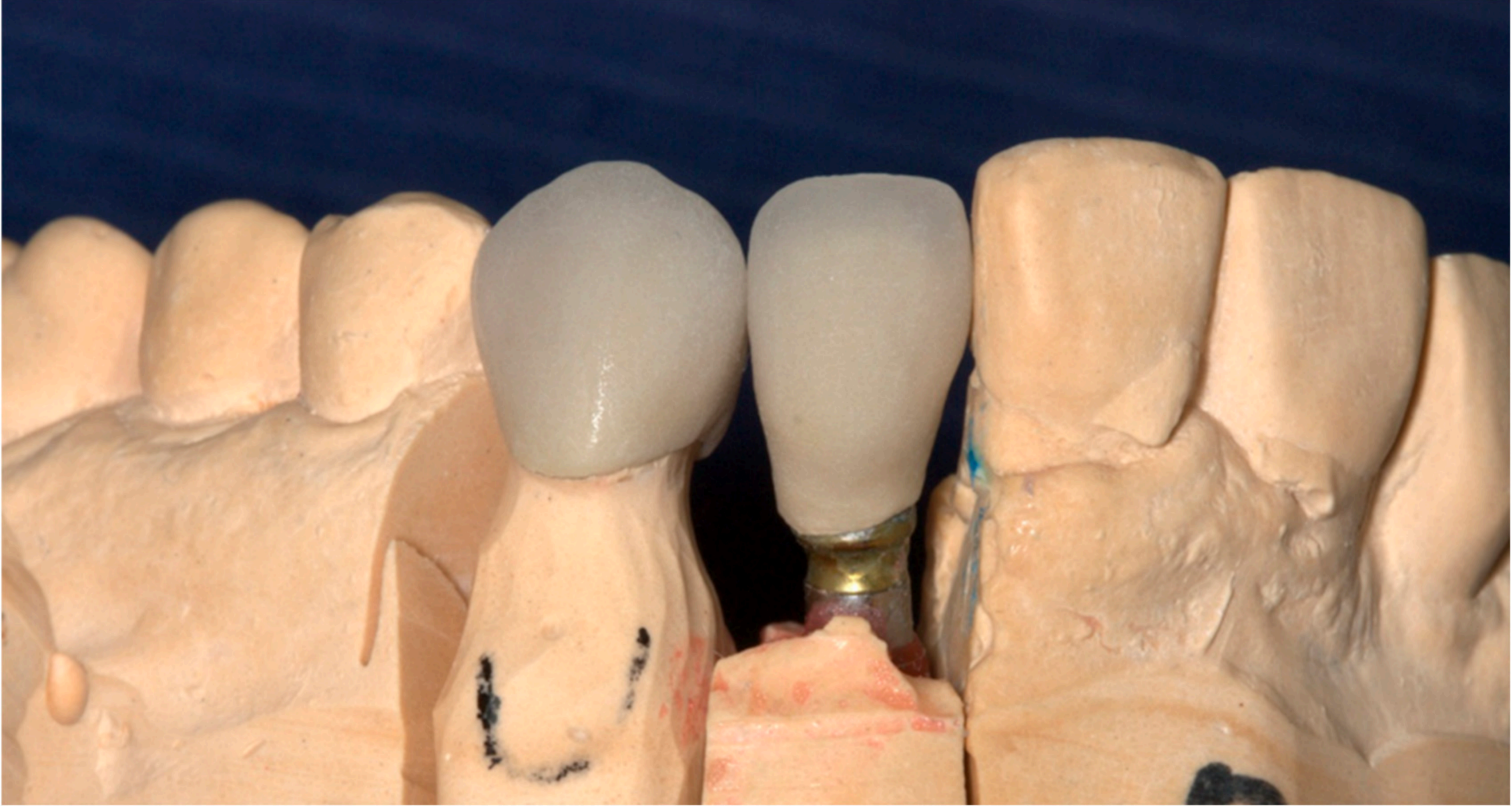
The restorations at the bisque-bake stage, on the mastercast.

**Figure 13:**
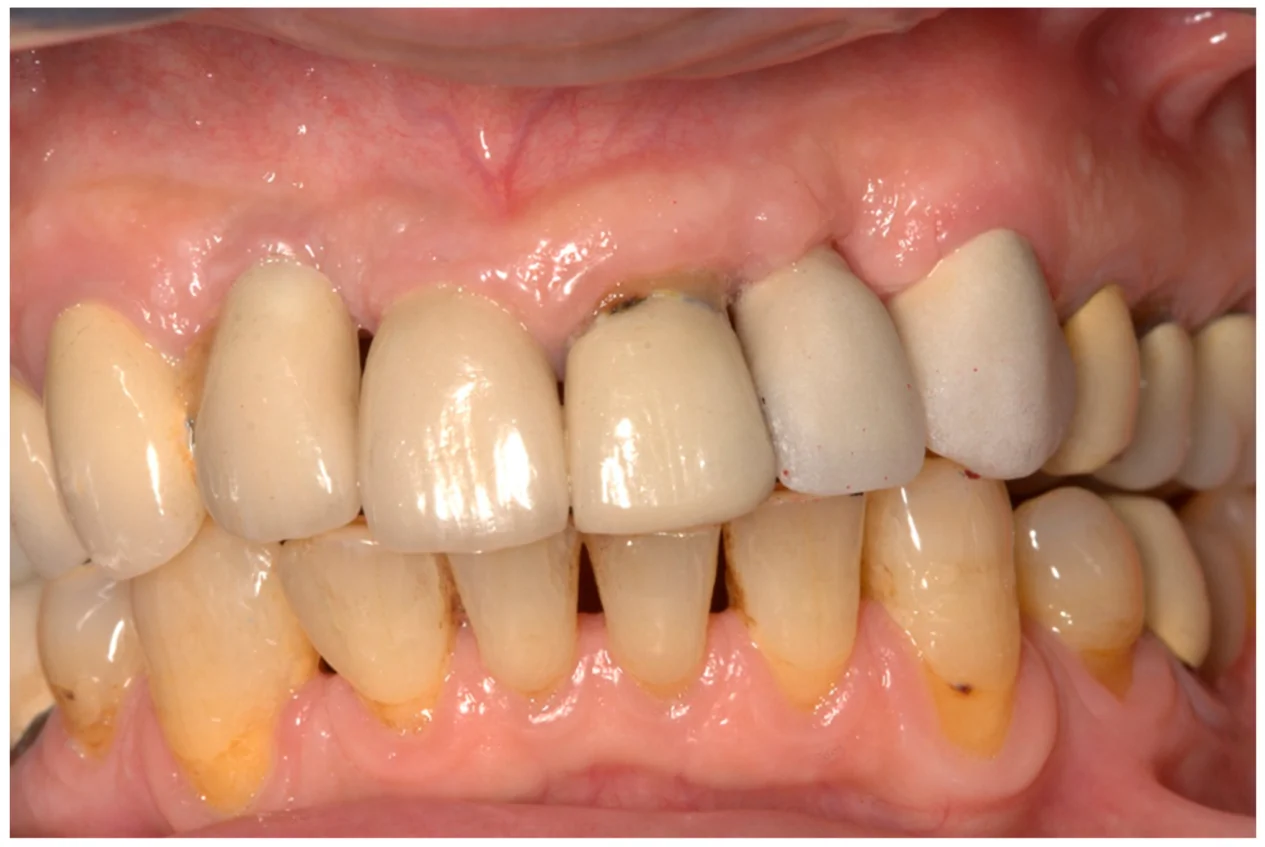
The bisque-bake try-in of restorations on teeth #22 and 23.

**Figure 14:**
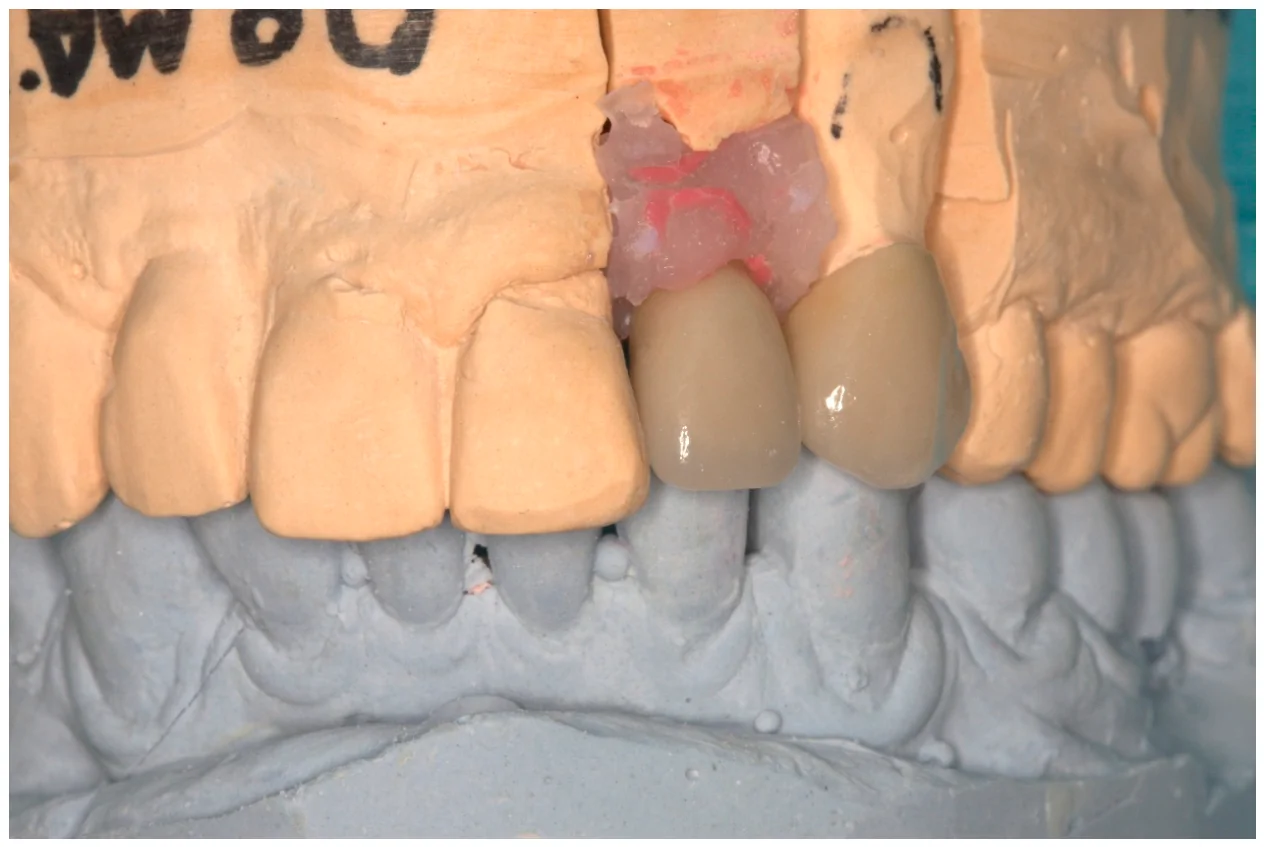
The glazed restorations on the mastercast.

**Figure 15:**
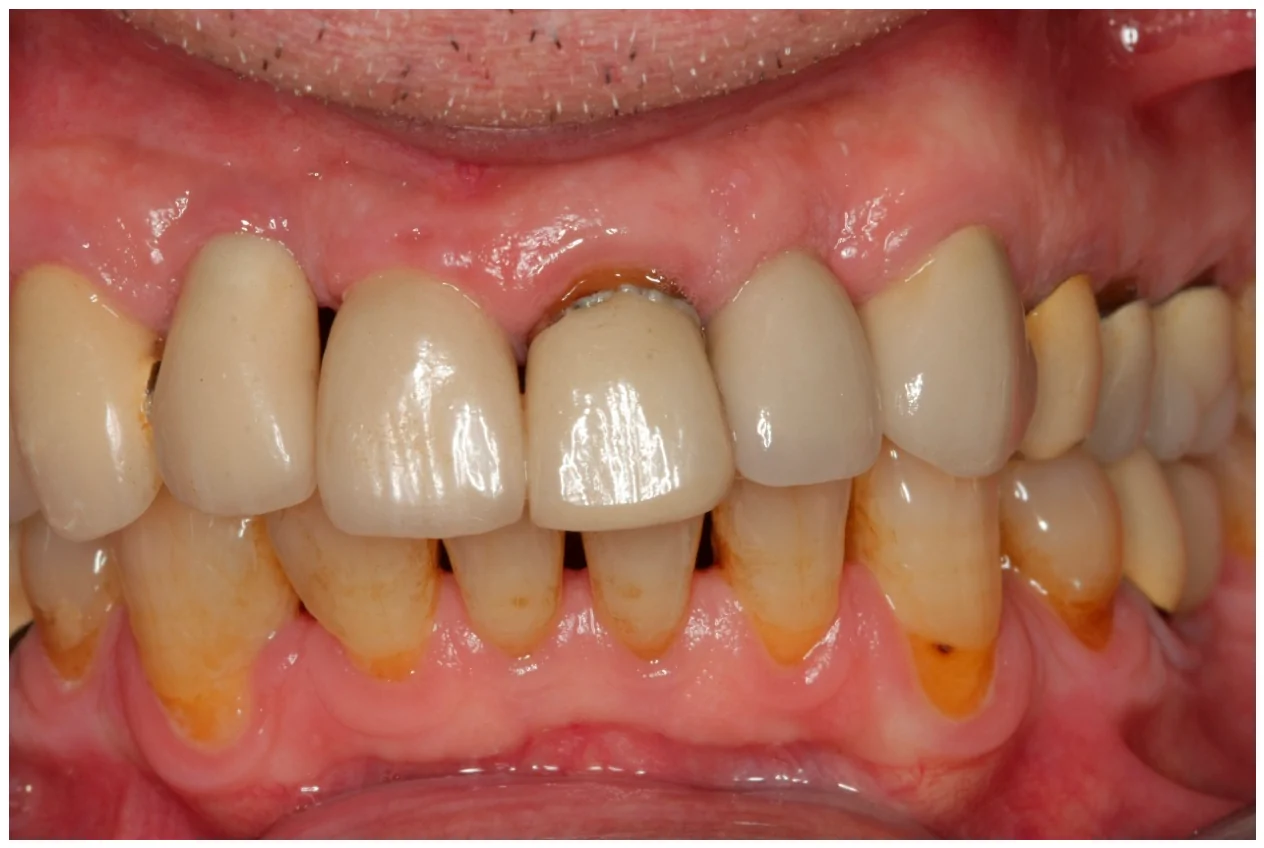
The cemented restorations, after maturation of soft peri-implant tissues.

Then the mastercast was sent to the dental lab, and the oxidation of the metal framework was performed; opaque porcelain was placed and fired, and finally, body as well as enamel porcelain were built and fired (Figure 12). Thereafter, the crowns were returned for the remaining clinical procedures, i.e., porcelain bake try-in (Figure 13), glazing at the dental lab (Figure 14), and final cementation with a permanent cement (GC Fuji I self-cured glass ionomer cement, GC Corp., Tokyo, Japan) (Figure 15). Before cementation, dental Teflon was placed in the screw access hole, over the screw, to facilitate retrievability. Excess cement was then removed.

## 3. Discussion

The use of a verification index is proposed as a standard of care in the rehabilitation of a complete arch, maxillary or mandibular, where 4 or more implants are inserted, since an increased number of implants is more prone to lead to impression discrepancies, which can induce more destructive consequences [2,3]. It is also used when an inaccuracy is detected, in order to reposition one or more implant analogs in the mastercast [43,44,45,46,47,48,49,50,51,52,53].

The inaccuracy in the impression was probably due to the different path of insertion of the impression post from that of the direction of the impression removal or/and rotation of the impression post during screwing the implant analog. Therefore, the implant analog should be cautiously attached to the impression coping by the restorative dentist in order to prevent or eliminate the possibility of rotation of the impression coping inside the impression material. A custom tray might have prevented a possible distortion of the impression, since it is more rigid than stock trays [34]. The impression material used around the impression coping was polyvinyl siloxane heavy body, which is considered less viscous and less accurate than polyether [26,34]. In contrast, no significant differences in impression accuracy were found in a systematic review between polyvinyl siloxane and polyether [28]. The single-stage double-mix technique is considered less accurate than monophase or two-stage techniques [38,39,40,41,42]. From a prosthodontic perspective, the use of the same low-viscosity impression material to accurately record both the finish line of the prepared tooth and the implant impression coping is generally recommended to optimize detail reproduction. Consequently, the reported impression technique appears to address a problem that may have been influenced, at least in part, by the selected impression protocol.

A small magnitude of discrepancy can be realistically corrected, up to 1–1.5 mm at the most, located at the incisal edge of the restorations, in order to have the required space for veneering porcelain [70,71] and at least 0.3 mm metal thickness [71,72]. In the case of a larger inaccuracy, the metal framework of the implant-supported crown may not be used. The existing implant abutment can still be used, following some preparation by the dental technician. The use of the verification index can also be used, in conjunction with the implant abutment, in case the inaccuracy is large enough. Then, the abutment should be ground in order to create adequate space for a new metal framework and veneering porcelain. In this case, the index should wrap around the abutment, but care should be taken not to interfere with the screw access hole. The screw access hole of the abutment can be filled with Teflon in order to prevent acrylic from getting inside the hole. In the case of a large misfit, the abutment may need to be prepared by the lab; therefore, the index can be sent to the dental lab, and this procedure can be done there as well.

The volumetric polymerization contraction of Pattern resin is reported to be 5% and was found to be lower than light-cured acrylic resins [73]. In order to reduce its effect, the acrylic resin was relined by adding a minimum amount, which corresponds to a minimum amount of dimensional change due to polymerization contraction. Alternatively, the verification index could be sectioned between the dies of prepared teeth and implant casting and reconnected with a minimal amount of acrylic resin.

In the present case report, the same removable die was used and repaired with PMMA, as in the reports by Moghadam et al. [44] and Hochstedler et al. [52], but these reports described the repositioning of removable dies for tooth-supported restoration. McCartney et al. [48], on the other hand, presented a sectioned acrylic resin framework pattern, connected intraorally, and replaced the implant analog in a fixed mastercast with the use of dental stone. The accuracy of working casts made by the direct impressions and acrylic verification coping procedures demonstrates similar discrepancies, less than 100 μm [74]. Therefore, this acrylic verification coping procedure is considered equally effective in working models’ fabrication. In the present study, repairing the removable die with stone would be very difficult to execute. It might also jeopardize the retention of the implant analog, since there was very little space for dental stone. Moreover, the acrylic resin can have mechanical retention in the stone irregularities of the removable die. In the present study, a Zeiser system was used for the fabrication of the mastercast, which is considered very accurate [62].

In the present study, metal-ceramic restorations were used. The use of metal-ceramic restorations is considered a gold standard in terms of flexural strength, compared to monolithic translucent zirconia or glass ceramics [66,67]. On the other hand, monolithic translucent zirconia or glass ceramics are considered more esthetic in terms of translucency [66,67,68,69], which can mimic that of natural teeth [68]. The existing anterior teeth were restored with metal-ceramic restorations, and therefore the new crowns were made with metal ceramics as well, in order to present similar esthetics to the existing ones.

This technique may represent an alternative to repeating the impression in selected clinical situations. Thus, the use of anesthesia for repeated impressions can be avoided. This technique allows for less discomfort for the patient, and reduction in the number of following appointments, and chair time. This is important in geriatric patients or patients with a moderate to severe gag reflex. It can also be used when no other impression post of this brand and type is available at the time. It can also lead to a reduction in cost, since the existing transmucosal abutment will be used, and possibly the existing metal framework of the restoration. This is feasible when the degree of inaccuracy is minimal. The patient, suffering from a moderate gag reflex, was happy not to have the impression made again, which would be frustrating for the patient as well as the restorative dentist.

The main drawback of this technique is that it is time-consuming and technique-sensitive, needing meticulous handling. This technique would be considered inappropriate if a dentist does not feel comfortable evaluating and adjusting the thickness of the metal framework, as well as the essential space for veneering porcelain. In this case, these procedures should be performed by the dental technician. Possible sources of error can be inaccuracy of the positioning of the verification index between intraoral and laboratory manipulation. Using dental loupes when manipulating the removable die is mandatory.

The technique described is an analog technique. Currently, the digital workflow in prosthodontics is very popular and can facilitate some of the procedures used in an analog workflow. In addition, the majority of dental practitioners are not using a digital workflow, and this technique is still applicable.

Mucogingival surgery was not performed, although it would lead to better esthetics in the gingival triangle area [75].

## 4. Conclusions

The use of the verification index and repositioning of the implant analog in the removable die may represent an alternative to repeating the impression in selected clinical situations. This is important for patients presenting moderate to strong gag reflexes, since the described procedure is less stressful. This is a single clinical case, and further clinical validation is necessary before broad clinical recommendations can be made.

## Data Availability

The original contributions presented in this study are included in the article. Further inquiries can be directed to the corresponding author.

## Abbreviations

The following abbreviations are used in this manuscript:

PFM	Porcelain fused to metal
PMMA	Polymethyl methacrylate
CBCT	Cone beam computed tomography
STL	Standard triangle language or standard tessellation language
